# Engineering constructed of high selectivity dexamethasone aptamer based on truncation and mutation technology

**DOI:** 10.3389/fbioe.2022.994711

**Published:** 2022-09-13

**Authors:** Yadi Qin, Yanan Qin, Hayilati Bubiajiaer, Fengxia Chen, Jun Yao, Minwei Zhang

**Affiliations:** ^1^ School of Pharmacy, Xinjiang Medical University, Urumqi, China; ^2^ College Life Science and Technology, Xinjiang University, Urumqi, China

**Keywords:** dexamethasone, truncation aptamers, mutation of aptamers, AuNPs, aptasensor

## Abstract

Various biosensors based on aptamers are currently the most popular rapid detection approaches, but the performance of these sensors is closely related to the affinity of aptamers. In this work, a strategy for constructed high-affinity aptamer was proposed. By truncating the bases flanking the 59 nt dexamethasones (DEX) original aptamer sequence to improve the sensitivity of the aptamer to DEX, and then base mutation was introduced to further improve the sensitivity and selectivity of aptamers. Finally, the 33 nt aptamer Apt-M13 with G-quadruplex structures was obtained. The dissociation constant (K_d_) was determined to be 200 nM by Graphene oxide (GO)-based fluorometry. As-prepared Apt-M13 was used for a label-free colorimetric aptamer sensor based on gold nanoparticles, the LOD was 3.2-fold lower than the original aptamer described in previous works. The anti-interference ability of DEX analogs is also further improved. It indicates that truncation technology effectively improves the specificity of the aptamer to DEX in this work, and the introduction of mutation further improves the affinity and selectivity of the aptamer to DEX. Therefore, the proposed aptamer optimization method is also expected to become a general strategy for various aptamer sequences.

## Introduction

Worldwide attention has been drawn to hormone residues and unlawful food additives. In clinical use, the synthetic glucocorticoid DEX provides potent anti-inflammatory properties (Nyman et al., 2020). As a growth promoter, DEX has been used in livestock production in the last century. Due to the strong endocrine-disrupting effect of DEX (Chen Z. et al., 2018; Mikiewicz et al., 2017), there are potential risks to human health. The use of DEX in livestock to promote growth has been outlawed by the European Union (Barbera, Biolatti, Divari, & Cannizzo, 2019). Meanwhile, dexamethasone and other hormones are also prohibited in most health care products. Consequently, the analysis and detection of DEX in food is an urgent task.

High-performance liquid chromatography (HPLC) and mass spectrometry (MS) are two common instrumental analytical techniques for detecting DEX with high sensitivity, accuracy, and precision (Guo et al., 2019; Chen et al., 2021). However, instrument-based analytical techniques demand high expenses for labor, time, and equipment. (Jia et al., 2020). Besides, DEX rapid detection methods mainly focus on immunological assays (Zhang et al., 2020). The accuracy of these methods mainly depends on the performance of the antibody. But antibodies could suffer low stability and the non-specific binding to analogs which do not meet the needs for on-site detection of DEX in food samples. Therefore, efficient and sensitive sensing configurations urgently need to be designed to detect DEX in food.

Aptamers can recognize target substances (eg. small molecules, proteins, cells, etc.) because they have a lot of receptor binding sites and spatial configurations that are simple to build, such as helices, hairpins, stem-loops, convex loops, etc (Wang, Chen, Larcher, Barrero, & Veedu, 2019; Ma et al., 2020). They have the characteristics of high stability, no immunogenicity, and easy synthesis and modification, therefore have been proposed as a substitute for conventional antibodies (Trinh et al., 2021; Zhang H. et al., 2017). However, the current aptamer-based small molecule sensing platforms are mostly designed using aptamers screened from SELEX, and the original aptamer length is 40–100 nt (Wang et al., 2022). Only a few fractions of bases in aptamer are involved in target binding (Chinnappan et al., 2019). In a tertiary conformation made up of the remaining unbound base, the target material may be interfered with. (Nie et al., 2018). To increase the affinity and specificity of aptamers, truncating non-binding base sequences has been thought of as a major method. (Svigelj et al., 2020; Azri et al., 2021). Additionally, it has been suggested to introduce some mutants by altering the nucleotides of aptamers. Surprisingly, some aptamer mutants have a higher binding affinity for the target substance as compared to the original aptamer (Biniuri et al., 2018; Khoshbin et al., 2019; Housaindokht, Izadyar, Bozorgmehr.,& Verdian, 2019). It's interesting to note that some screened aptamers with G-quadruplexes have higher specificity and sensitivity (Wei and Zhang, 2018; Khoshbin et al., 2020). The G-quadruplex (G4) consists of guanine-rich sequences that can form chair-like parallel structures and are stabilized with ligands in the form of Hoogsteen hydrogen bonds through guanine tetrads (Nao et al., 2020; Liu et al., 2021). Some corticosteroids have been demonstrated to form stable complexes with their G4 aptamers (Huang et al., 2018).

In this study, the redundant base of the original aptamer was truncated to improve the sensitivity of colorimetric sensors based on AuNPs. To further improve affinity, the truncated aptamer was then subjected to multiple base mutations to induce the formation of a G-quadruplex structure. A 33 nt mutant aptamer Apt-M13 was obtained with significantly improved sensitivity and specificity compared to the original aptamer sequence. The Apt-M13 obtained in this study has great potential in sensing configurations combined with other nanomaterials for rapid DEX detection.

## Materials and methods

### Materials and reagents

All aptamers and SYBR Green I (SGI) were synthesized by Sangon Biotech Co., Ltd. (Shanghai, China)., and the sequences were shown in Supplementary Table S1. Dexamethasone (DEX), hydrocortisone (HC), prednisone acetate (PDN), budesonide (BUD), and chloramphenicol (CHL) were obtained from Macklin Biochemical Co., Ltd. (Shanghai, China). Methanol (CH_3_OH) and ethyl acetate (C_4_H_8_O_2_) were obtained from Tianjin Beilian Fine Chemical Development Co., Ltd. (Tianjin, China). Purified water (Wahaha Group Co., Ltd. Hangzhou, China) was throughout used as a solvent for colorimetric analysis and fluorescence titration for Kd measurement. Raw milk samples were purchased at local markets. All experiments were repeated 3 times, drawing charts and calculating the standard deviations (error bars) of three groups of experimental data through Origin software.

### Instrumentation

UV absorption spectra were recorded at wavelengths of 400–800 nm using a SHIMADZU UV-2700 UV-vis spectrophotometer (SHIMADZU Ltd., Japan). The form and size distribution of Au NPs was determined in a JEM-1230(JEOL Co., Japan) transmission electron microscope (TEM) determination. Allegra-64R centrifuge was used for the centrifugation (Beckman Coulter, Inc. United States). The ultrasonic treatment was carried out on a KQ3200DE CNC ultrasonic cleaner (Kunshan Instrument Co., China). ZHWY-2102C Incubator shaker (Shanghai Zhicheng Analytical Instrument Manufacturing Co., Ltd. China) was used for 37°C shaking incubation. Fluorescence intensity was measured by an Infinite E Plex microplate reader (Tecan Trading Co., Ltd. Austria). Atomic force microscopy (AFM) imaging was obtained using a Dimension Icon system (Bruker AXS Co., Germany). The fluorescence spectra of samples were performed by using an RF-5301PC spectrofluorophotometer (Shimadzu, Japan) at room temperature.

### Truncation and mutation of aptamers

The truncated aptamers were obtained by truncating the part of the bases in the original sequence (Mehennaoui et al., 2019) that was not involved in specific binding based on molecular docking analysis. The optimal truncated aptamers were given nucleotide mutation in which the bases surrounding the G bases were adjusted, causing the mutant aptamer to take the shape of a G-quadruplex. Additionally, determine whether the mutant sequence creates a G-quadruplex structure using the G-quadruplex analysis tool (QGRS Mapper). All aptamers’ secondary structures were predicted using the Mfold website (www.unafold.org/mfold/applications/dna-folding-form.php). The dexamethasone’s three-dimensional structure was obtained from the PubChem database (https://pubchem.ncbi.nlm.nih.gov/compound/5743). The three-dimensional structure of aptamer was first compiled into BPSEQ format through secondary structure, converted into a dot-bracket format, and then predicted by nucleic acid structure modeling server RNAcomposer (https://rnacomposer.cs.put.poznan.pl/). Utilizing AutoDock 4.2 tools, polar hydrogen atoms, Kollman atom type charges, and solvation parameters can be added to the aptamer. Lattice modules of size 120 × 120 × 120 with intervals of 0.375 were employed in the calculation. The final docked structure was assessed based on the binding free energy after 100 independent docking runs and calculations using the Lamarckian evolutionary algorithm with a maximum of 25 million energy assessments. Pymol 2.0 was used to process the visualization of docking findings.

### Fluorescence measurement of dissociation constants (K_d_)

The determination method of the K_d_ value refers to the previous study (Chen et al., 2014). Take 100 μL of aptamer solutions of different concentrations (10–250 nM), denature at 95°C for 10 min, and immediately take an ice bath for 10 min. Add 100 μL of DEX solutions (1.5 μM) and incubate at 37°C for 2 h with shaking. Then, GO with the optimal mass ratio (the mass ratio of GO to aptamer is 200:1) was added and incubated for 30 min with shaking to adsorb ssDNA that was not bound to the target. Centrifuge at 13,000 r/min for 15 min at 4°C, measure the fluorescence intensity of the supernatant (excitation wavelength 485 nm, emission wavelength 520 nm) with an Infinite E Plex microplate reader, and use sterile water instead of the target as the negative control. The relative fluorescence intensity (ΔF,△F = F - F_0_) is fitted to the nonlinear saturation binding curve of different concentrations of aptamer by Origin 9.0 software, F is the fluorescence intensity of the experimental group; F_0_ is the fluorescence intensity of the negative control group. The dissociation constant (K_d_ value) was calculated by the formula:

$$\Delta F\;=\;\mathrm{Bmax}\;*\;\mathrm{ssDNA}/\left(K_{d}+\mathrm{ssDNA}\right)$$

Bmax is the maximum fluorescence intensity, and ssDNA is the added aptamer concentration.

Finally, the aptamer forms a G quadruplex structure was verified using SGI dye. The aptamer with a concentration of 50 nM was denatured at 95°C for 10 min and then cooled down to room temperature for 15 min. Then, 100 *µ* L aptamer solution was mixed with 15 *µ* L 1X concentration of SGI for 8 min. The mixture was diluted with water to a volume of 500 μL after a homogeneous mixing process. Finally, the fluorescence spectrum of the solution was recorded with a fluorophotometer, and set the parameters as follows: the excitation wavelength was 495 nm; the fluorescence intensity at 510–600 nm was scanned; the excitation slit was 5 nm; the emission slit was 5 nm.

### Detection of DEX by the colorimetric sensor

300 μL of AuNPs were mixed with 200 μL (0.07 μM) of various aptamers, vortexed for 30 s, and incubated at room temperature for 30min. Then 200 μL of DEX solutions at various concentrations were added into the mixture, vortex for 30 s, let to react for 30 min, and finally added with 200 μL (36.7 mM) of NaCl solution and mixed well, The final mixed solution volume is 900 μL. The UV absorption spectrum of the final solution was measured by a scanning wavelength range of 400–800 nm, and the color variations of the AuNPs solution were recorded. Each experiment was performed three times.

### Selectivity assay

To investigate the DEX detection selectivity of mutant and truncated aptamers in DEX detection. The aptamers were mixed with DEX analogs including prednisone (PDN), hydrocortisone (HC), Budesonide (BUD), and chloramphenicol (CHL) respectively, and added to the DEX colorimetric sensor. The absorbance ratio ΔR (A_650_/A_520_) was recorded and ΔR = Absorbance ratio A_650_/A_520_ containing DEX analog - Absorbance ratio A_650_/A_520_ of blank solution.

### Pretreatment of milk samples

2 g of raw milk were mixed with 30 ml of ethyl acetate, vortexed for 30 s, and sonicated for 15 min, the mixed solution was centrifuged at 4500 rpm for 15 min to remove the protein and fat, finally, the supernatant was taken and transferred to the hydrophile lipophile balance **(**HLB) extraction cartridges for further purification, and the filtrate was collected and dried under nitrogen at 40°C. The residue was reconstituted with a methanol-H_2_O solution (30%, v:v) as the test sample.

## Results and discussion

### Characterization of AuNPs and principle of colorimetric sensor

**FIGURE 1:**
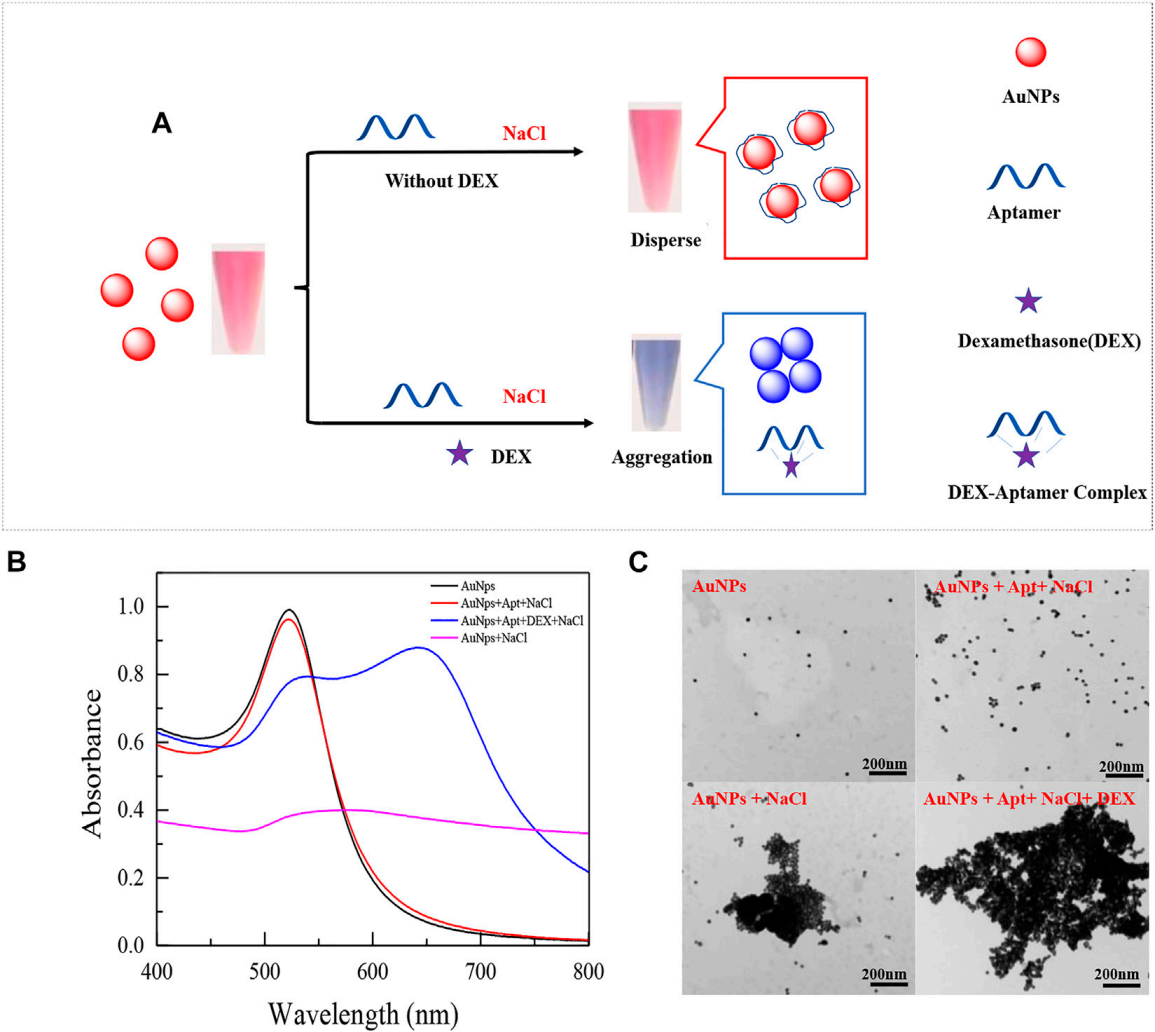
Schematic diagram of an aptamer-based colorimetric sensor **(A)**. UV absorption spectra of prepared AuNPs mixed with different solutions **(B)**, the UV spectrum shown is: (black line) 300 μL AuNPs (13 nm) only; (red line) 300 μL AuNPs (13 nm) + 200 μL 0.07 μM aptamer +200 μL 36.7 mM NaCl; (blue line) 300 μL AuNPs (13 nm) + 200 μL 0.07 μM aptamer +200 μL 350 nM DEX+ 200 μL 36.7 mM NaCl; (purple line) 300 μL AuNPs (13 nm) +200 μL 36.7 mM NaCl. And their respective TEM images **(C)**.

The average diameter of the synthesized AuNPs is about 13 nm, the preparation method is consistent with the previous study (Qin, Bubiajiaer, Yao, & Zhang, 2022), and the concentration of AuNPs is calculated by Beer-Lambert law as 3.67 nM. The principle of the designed colorimetric sensor is shown in Figure 1. The sensor is based on the specific binding of aptamer and DEX to induce the aggregation of AuNPs in presence of a high concentration of NaCl. In the absence of DEX, aptamers were attached to the surface of AuNPs to resist NaCl-induced aggregation (Figure 1B red line). When DEX is contained in the system, aptamers preferentially form stable complexes with DEX, resulting in AuNPs being exposed and aggregated under the action of a high concentration of NaCl (Figure 1B blue line). Based on the above, it is obvious that the performance of colorimetric sensors is closely related to the affinity of aptamers to DEX. Therefore, the affinity of truncated or mutant aptamers for DEX was assessed by changes in colorimetric sensor sensitivity and specificity.

### Truncation and characterization of the aptamers

**FIGURE 2:**
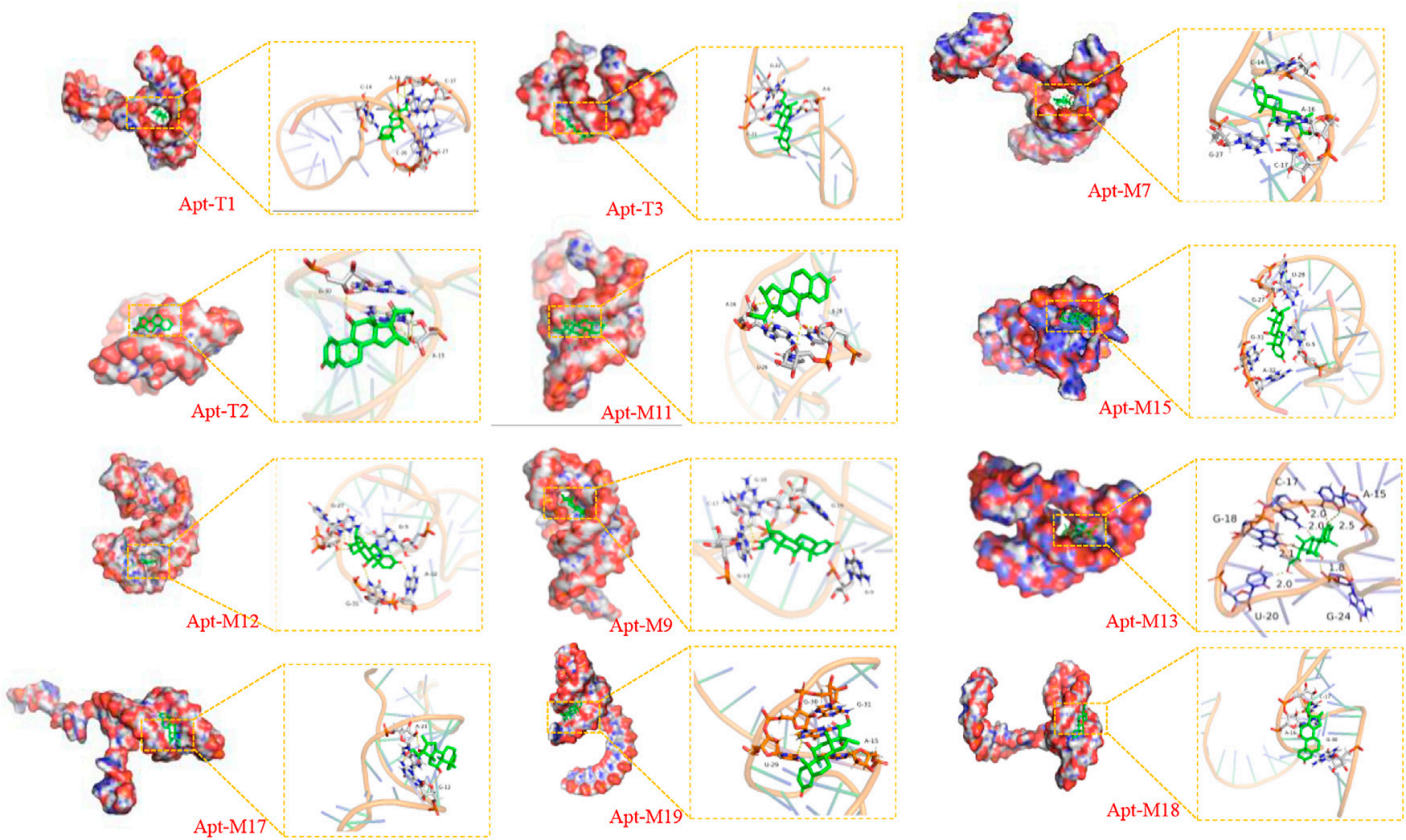
Visualization of molecular dynamics simulations of truncated aptamers and mutated aptamers with DEX.

The bases of the original sequence were obtained through 19 rounds of screening with dexamethasone conjugated to agarose 6 beads (Mehennaoui et al., 2019). The bases that were primarily responsible for DEX’s specific binding were obtained through molecular docking and molecular dynamics simulation, as shown in Supplementary Figure S1, which is primarily focused on the two stem-loop structures (Qin, Bubiajiaer, Yao, & Zhang, 2022). Specific secondary structures facilitated the binding of target substances (Albada, Golub, Willner, 2015). To improve the affinity of the aptamer for DEX, the original 59 nt aptamer Apt1 was truncated. Firstly, the terminal branches on both sides of Apt1 that were not involved in specific binding were truncated and retained two stem-loop and stem-loop intermediate connecting regions to obtain the 41 nt truncated aptamer Apt-T1 (Supplementary Figure S1 *Step1*). Secondly, based on the Apt-T1, two stem-loops were kept and the binding region between the two stem-loops was truncated to obtain a 33 nt truncated aptamer Apt-T2 (Supplementary Figure S1 *Step2*). Finally, it is found that most of the binding sites were concentrated in stem-loop B, so the stem-loop A was truncated, and the stem-loop B was kept to get a 24 nt truncated aptamer Apt-T3 (Supplementary Figure S1 *Step3*). To explore changes in truncated aptamers, the secondary structure of the truncated aptamer was predicted by the Mfold server (Supplementary Figure S1), and the molecular docking analysis of these aptamers and DEX was performed (Figure 2 and Table 1). The results show that the binding energies of the truncated aptamers and DEX were higher than the original aptamer, which were −6.94, −8.17, and −7.24 kcal*mol^−1^. This theoretically proves that truncating redundant bases helps to improve the affinity of the aptamer. To further verify the sensitivity and affinity of the truncated aptamer, the original aptamer and the truncated aptamers were used for the established colorimetric sensor.

**TABLE 1:** Linear responses and molecular docking results of each aptamer.

	Number of bases	G-quadruplex	Regression Equation(y = a+b*x)	Standard Error(b)	Coefficient (*R* ^2^)	Linear range (nmol/ml)	LOD (nmol/ml)	Binding energy (kcal/mol)
Apt1	59	-	y = 0.00222x+0.208	6.88*10^–5^	0.997	10–350	0.5	−5.64
Apt-T1	41	-	y = 0.00387x+0.3015	1.78*10^–4^	0.993	2–250	0.28	−6.96
Apt-T2	33	-	y = 0.00479x+0.173	1.49*10^–4^	0.997	2–200	0.23	−8.17
Apt-T3	24	-	y = 0.00412x+0.275	5.47*10^–5^	0.999	2–250	0.27	−7.24
Apt-M7	33	Yes	-	—	-	-	-	−7.02
Apt-M9	33	Yes	y = 0.00408x+0.207	2.43*10^–4^	0.988	2–250	0.27	−7.78
Apt-M11	33	Yes	y = 0.00333x+0.207	7.78*10^–5^	0.999	10–250	0.33	−6.88
Apt-M12	33	Yes	y = 0.00499x+0.182	2.64*10^–5^	0.999	2–200	0.22	−8.27
Apt-M13	33	Yes	y = 0.00716x+0.222	3.5*10^–4^	0.994	1–100	0.15	−9.12
Apt-M15	33	Yes	y = 0.00477x+0.151	1.03*10^–4^	0.998	2–200	0.23	−8.07
Apt-M17	33	Yes	-	—	-	-	-	−6.29
Apt-M18	33	Yes	-	—	-	-	-	−5.56
Apt-M19	33	Yes	-	—	-	-	-	−7.27

**FIGURE 3:**
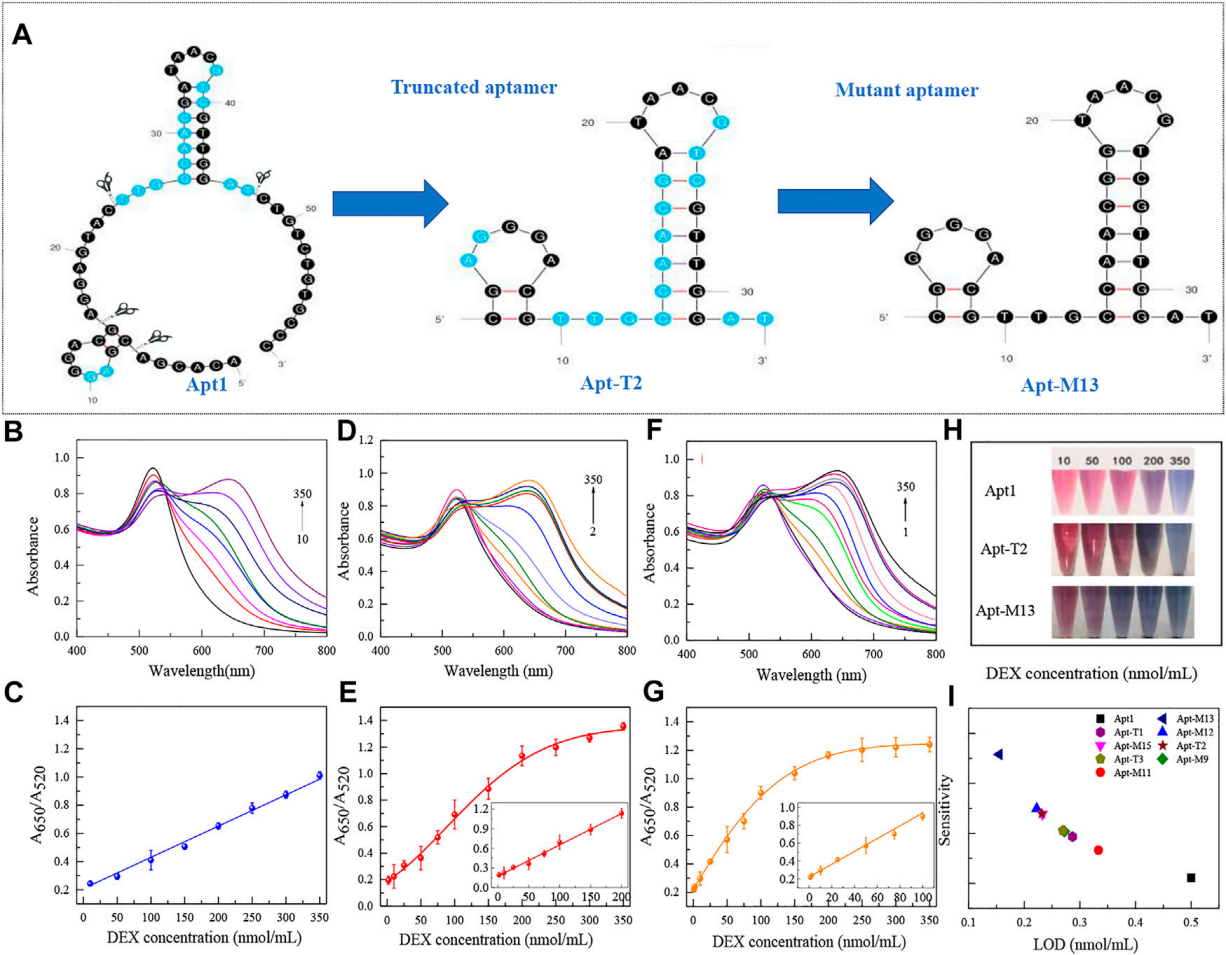
Secondary structure of the original sequence Apt1, and the corresponding truncated and mutant aptamers **(A)**. UV absorption spectra of original aptamer-based sensing systems at DEX concentrations of 10–350 nmol/ml **(B)** and linear responses at different concentrations **(C)**. UV absorption spectra of truncated aptamer-based sensing systems at DEX concentrations of 2–350 nmol/ml **(D)** and linear responses at different concentrations **(E)**. UV absorption spectra of mutant aptamer-based sensing systems at DEX concentrations of 1–350 nmol/ml **(F)** and linear responses at different concentrations **(G)**. Color responses of three aptamers at different DEX concentrations **(H)**. Comparison of analytical performance based on each aptamer in a colorimetric sensor **(I)**.

Usually, the key of the colorimetric method is the specific binding of aptamer with the target substance. Therefore, the detection performance of the colorimetric sensors was used to directly reflect the binding strength of different aptamer sequences to DEX. Under the identical experimental parameters, the LOD of each aptamer on the colorimetric sensor was verified using DEX at a series of concentrations from 1 to 350 nmol/ml. The results were shown in Figure 3 and Table 1. Truncating the redundant sequence can effectively increase the aptamer’s affinity for DEX, as shown by the enhanced LOD of the colorimetric sensor based on the truncated aptamer Apt-T1 (Supplementary Figure S2A).compared to the original sequence (Figures 3B,C). The aptamer Apt-T2 obtained by further truncating the aptamer Apt-T1 exhibited a higher DEX affinity 2.2-fold that of the original sequence, DEX was detected down to 0.23 nmol/ml (Figures 3D,E). Although the truncated aptamer Apt-T3 is efficient compared to the original sequence, the LOD (0.27 nmol/ml) of the sensor is higher than that of Apt-T2 (Supplementary Figure S2B). This indicated that the two stem-loop structures of the original aptamer were essential in the specific binding of DEX. The experimental results were consistent with the changes in the binding energy of different aptamers with DEX obtained by the molecular docking.

**FIGURE 4:**
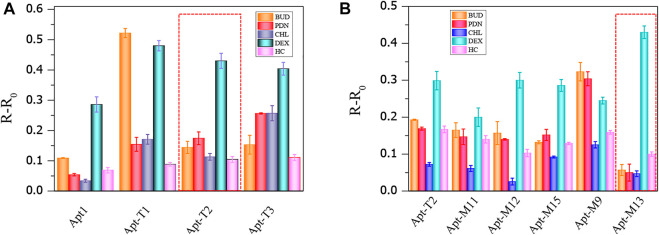
Cross-reactivity of truncated aptamer sensors to 100 nmol/ml DEX and analogs **(A)**, cross-reactivity of mutant aptamers to 50 nmol/ml DEX and analogs **(B)**.

Although truncation strategies have been proved to be effective in enhancing the sensitivity of aptamers to DEX by colorimetric sensors, the change of aptamer structure may also lead to changes in specificity to DEX. The result is shown in Figure 4A, the truncated aptamer Apt-T2 showed similar specificity characteristics to the original aptamer Apt1, with a significant increase in A_650_/A_520_ in DEX samples, while other analogs did not lead to a significant increase in A_650_/A_520_. However, the truncated aptamers Apt-T1 and Apt-T3 have greater cross-reactivity with DEX analogs. Apt-T1 is even more selective for BUD than DEX. Although the aptamer obtained by the truncation strategy has better sensitivity, the specificity still needs further improvement.

### Mutation and characterization based on the truncated aptamer Apt-T2

To further improve the sensitivity and specificity of aptamers for DEX, base mutations were introduced based on the truncated aptamer Apt-T2. The principle is to mutate the non-G-base around the G-base of the Apt-T2 sequence to G-base while keeping the double stem-loop structure of the aptamer and further induce the aptamer to form a G-quadruplex structure. Firstly, only one non-G base was altered in the Apt-T2 sequence. Fourteen sequences (Apt-S1 to Apt-S14) were obtained as shown in Supplementary Table S2. Unfortunately, The obtained sequences could not form a G-quadruplex structure after being analyzed by the QGRS Mapper online prediction platform. Following this, we attempted multisite mutagenesis, nineteen aptamer sequences were obtained as shown in Supplementary Table S2. As expected the aptamer Apt-M7, Apt-M9, Apt-M11, Apt-M12, Apt-M13, Apt-M15, Apt-M17, Apt-M18, Apt-M19 could generate G-quadruplex structures (Table 1). More importantly, the sequences of Apt-M 9, Apt-M11, Apt-M12, Apt-M13, and Apt-M15 could maintain the double stem-loop structure while forming G-quadruplex (Supplementary Figure S3). The binding energies were −7.78, −6.88, −8.27, −9.12, and −8.07 kcal*mol-1, respectively, higher than the mutant aptamer sequence with only a single stem ring structure. This further illustrates the importance of double stem ring structure in a specific binding with DEX (Table 1).

In addition, the binding conformation of Apt-M13 with the maximum binding energy was analyzed. Detailed molecular interaction analysis shows that dexamethasone is composed of multiple six-membered carbon rings and has strong hydrophobicity. It has a certain hydrophobic interaction with aptamer, which helps to stabilize the binding of small molecules to sites. Meanwhile, the hydroxyl group and the carbonyl group of DEX can also form hydrogen bonds with the active groups of G-18, A-15, G-24, and U-20 bases of the aptamer, with hydrogen bond distances of 2.1 Å, 2.25 Å, and 1.8 Å respectively. In addition, the fluorine atom of DEX forms halogen bonds with the C-17 base, which plays a significant role in forming stable complexes with the aptamer. In combination with Figure 2, we found that DEX was well wrapped in the pocket formed by the aptamer and was not easy to leave.

The sensitivity and affinity of Apt-T2-based mutant aptamers Apt-M9, Apt-M11, Apt-M12, Apt-M13, and Apt-M15 were investigated in colorimetric sensors under the same experimental parameters. The results were shown in Table 1 and Supplementary Figure S4. Compared with the truncated sequence Apt-T2, the Apt-M13-based colorimetric sensor’s LOD increased by roughly 1.5 times (Figures 3F,G). The LOD of the colorimetric sensor based on Apt-M12 and Apt-M15 were comparable or somewhat enhanced, the colorimetric sensor based on Apt-M9 and Apt-M11, however, has a worse LOD. In addition, the color response of the original aptamer Apt1, the truncated aptamer Apt-T2, and the mutant aptamer Apt-M13 were observed in the colorimetric sensor to visually reflect the binding strength of the aptamer with the DEX. when the DEX concentrations were 200, 100, and 50 nmol/ml respectively, the color change of AuNPs was visible to the naked eye, which indicated that the relative signal of Apt-M13 was higher than that of other aptamers (Figure 3H).

Then, the specificity of Apt-T2-based mutant aptamers was investigated. Given that the linear range of the colorimetric sensor based on the aptamer Apt-M13 is 1–100 nmol/ml the concentration of DEX and interfering compounds were adjusted to 50 nmol/mL. As shown in Figure 4B, compared with other aptamers, the specificity of Apt-M13 is highly specific to DEX and has insignificant cross-reactivity with its analogs. According to the results above, the mutant aptamer M13’s sensitivity and specificity have both increased. Combining all results, the aptamer Apt-M13 exhibited the highest affinity for DEX (Figure 3I).

### GO-based fluorescence detection of aptamer affinity

**FIGURE 5:**
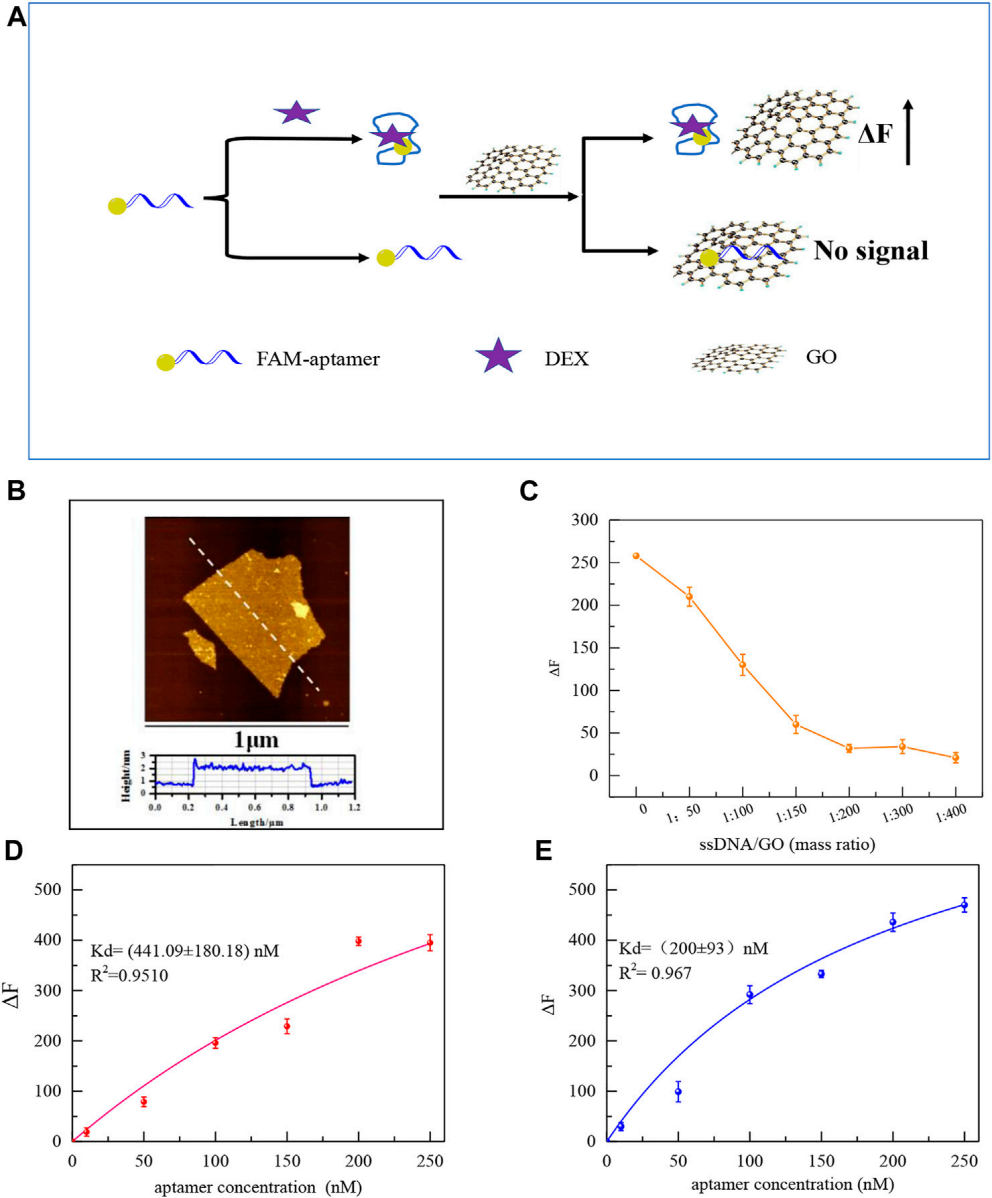
Schematic diagram of the principle of affinity fluorescence detection of GO-based aptamers for DEX **(A)**. The AFM images of GO **(B)**. Effect of GO addition concentration on ΔF **(C)**. Saturation curves of original aptamers for DEX by GO fluorescence method (100 μL original aptamer +100 μL 1.5 μM DEX+ the mass ratio of GO to aptamer is 200:1 **(D)**, and saturation curves of mutant aptamers (100 μL aptamer Apt-M13 + 100 μL 1.5 μM DEX+ the mass ratio of GO to aptamer is 200:1) **(E)**.

The improved performance of the colorimetric sensor may be attributed to the reduced non-characteristic absorption of the aptamer on the surface of AuNPs after eliminating redundant bases. GO and FAM modified Aptamers were employed to further investigate the variations in the affinity of aptamers for DEX. The principle to test the affinity of aptamers is shown in Figure 5A, GO’s 2D surface structure and excellent energy transfer capability enable it to strongly adsorb biomolecules through π-π stacking or hydrogen bonding interactions and to bind nearby fluorescent substances through a fluorescence resonance energy transfer (FRET) mechanism. The fluorescence was quenched. (Zhang Z. et al., 2017). As shown in Figure 5A, after the aptamer labeled with the FAM group at the 5′ end was incubated with the DEX, the aptamer bound to the DEX formed a three-dimensional conformation, which could not be stably adsorbed and bound to GO, and the fluorophore would not be quenched. Aptamers not bound to DEX are stably adsorbed on the surface of GO resulting in fluorescence quenching. After centrifugation, the supernatant is the target-ssDNA complex, and the affinity (K_d_ value) of aptamer with DEX can be effectively calculated by measuring its fluorescence intensity.

The stable monolayer GO was synthesized in accord with our previous study, which plays a significant role in the adsorption of the aptamer (Meng et al., 2022). Atomic force microscopy(AFM) shows that the thickness of the GO sheet is 1.7 nm (Figure 5B). To ensure GO can fully adsorb free aptamers, as shown in Figure 5C, with the increase of GO, the fluorescence intensity decreased. When the mass ratio of GO to aptamer was 200:1, the aptamer was adsorbed by GO which made the fluorescence quenched. Then, the affinity of the mutant aptamer Apt-M13 and the original aptamer sequence Apt1was compared by the GO fluorescence method. It can be seen in Figures 5D,E that the K_d_ value was (200 ± 93) and (441 ± 180) nM, respectively. The smaller the K_d_ value, the stronger the affinity of the sequence. It is demonstrated that the affinity of the mutant aptamer Apt-M13 has been significantly improved.

Interestingly, the G-quadruplex structure can be bound by some fluorescent dyes to produce strong fluorescence, such as Thioflavin T (ThT) (Kadam et al., 2022; Trinh et al., 2022) and SGI (Chen X. et al., 2018; Li, Tu & Luo., 2018). A simple SGI dye experiment was used to verify that Apt-M13 formed a G-quadruplex structure. As shown in Supplementary Figure S5, Apt-M13 aptamer could interact with SGI to generate strong fluorescence signals. SGI is a selective intercalation dye, which can bind to the single-stranded DNA like an aptamer with a G-quadruplex structure and generate strong fluorescence emission, while it does not produce an obvious fluorescence signal when it binds to the single-stranded DNA of other structures (Yi et al., 2019). It is proved that aptamer Apt-M13 forms a G quadruplex structure, which is also consistent with the prediction results of the QGRS server.

### Detection of DEX in raw milk

The feasibility of analyzing DEX in real samples was investigated using a colorimetric sensor of the mutant aptamer Apt-M13. the sample matrix cleansed by HLB extraction cartridges will scarcely have an impact on the colorimetric sensor. 20, 50, and 100 nmol/ml of DEX were added to raw milk to evaluate the performance of the designed aptasensor to detect DEX. The experimental results were summarized in Table 2, the recovery values were in the range of 97.88–110.05% and the relative standard deviation (RSD) of this sensor was from 2.87 to 5.13%. It shows that the developed colorimetric sensor may be applied to the determination of DEX in food. Compared with other reported DEX sensors (Table 3), established colorimetric sensors have the advantages of quick detection without specialized equipment and trained operators and can avoid the drawbacks of antibody-based detection, such as a high rate of false positives. Meanwhile, the synthesis cost of the truncated aptamer sequence is lower than the original aptamer, which meets the requirement of high-throughput and low-cost rapid detection. Therefore, the designed DEX colorimetric sensor has great potential for DEX detection in food.

**TABLE 2:** Analysis of results of DEX in raw milk.

Sample	Added(nmol/mL)	Found(nmol/mL)	Recovery(%)	RSD(%)
Milk	20	22.01	110.05	5.13
	50	48.94	97.88	4.51
	100	103.25	103.25	2.87

**TABLE 3:** Comparable methods for determination of dexamethasone.

Method	Linear range	LOD	Applications	Recovery	Ref.
Electrochemical aptasensor	2.5–100 nM	2.12 nM	tab water and drinking water	81.5–103.2%	Mehennaoui et al. (2019)
Immunochromatographic assay	0.05–5 ng/ml	0.58 ng/ml	Muscle and liver	84.5%	Zhang et al. (2019)
Electrochemical sensor	0.05–30 mM	3.0 nM	human urine and serum samples	97.0–102.0%	Fatahi et al. (2017)
LC-MS/MS	2.5–500 ng/ml	2.5 ng/ml	nude mice plasma	-	Yuan et al. (2015)
First and third derivative spectrophotometry electrode	0.25–50.0 μg/ml	0.1 μg/ml	Urine and Serum	93.3–108%	Montazeralmahdi et al. (2019)
Colorimetric biosensor	0.1–9 ng/ml	2.0 μg/kg	food supplements and cosmetic samples	–	Zhang et al. (2020)
Electrochemical sensor	10–500 μg/ml	6 μg/ml	herbal medicines	-	Primpray et al. (2019)
Lateral flow immunoassays	-	0.3 ng/ml	milk and pork	80.0–122.8%	Li et al. (2021)
Colorimetric biosensor	1–100 nM	0.15 nM	milk	97.88–110.05%	This assay

## Conclusion

In summary, based on the results of molecular docking and molecular dynamics simulation, the high-sensitive aptamer was obtained through the original aptamer sequence of DEX was effectively truncated and the base mutation was introduced. The mutant aptamer Apt-M13 exhibited a significant affinity for DEX, and with a 3.2-fold lower LOD than the original ligand sensor. In addition, the mutant aptamer Apt-M13 has been used to spike the sensor with recoveries ranging from 97.88 to 110.05% in milk samples, indicating a great potential for rapid detection of DEX in food. Meanwhile, the aptamer truncation and mutation strategy used in this study can effectively improve the affinity and selectivity of aptamers to target substances, which also provides a reference for the optimization design of aptamers in the future.

## Data Availability

The original contributions presented in the study are included in the article/Supplementary Material, further inquiries can be directed to the corresponding authors.
