# Supplementary material for: Engineering constructed of high selectivity dexamethasone aptamer based on truncation and mutation technology
Source: Front Bioeng Biotechnol. 2022 Sep 13;10:994711. doi: 10.3389/fbioe.2022.994711 (PMC9513367; doi:10.3389/fbioe.2022.994711)
Supplement: Supplementary file 1 [file DataSheet1.docx]

Supporting Information for

| **Aptamer** | **Sequence** |
| --- | --- |
| Apt1 | 5′-ACA CGA CGA GGG ACG AGG AGT ACT TGC CAA CGA TAA CGT CGT TGG ATC TGT CTG TGC CC-3′ |
| Apt-T1 | 5′-CGA GGG ACG AGG AGT ACT TGC CAA CGA TAA CGT CGT TGG AT-3′ |
| Apt-T2 | 5′-CGA GGG ACG TTG CCA ACG ATA ACG TCG TTG GAT-3′ |
| Apt-T3 | 5′-TTG CCA ACG ATAACGTCGTTGGAT-3′ |
| Apt-M9 | 5′-CGA GGG ACG TTG GCA ACG GTA ACG TCG TTG GAT-3′ |
| Apt-M11 | 5′-CGA GGG ACG TTG CCA ACG GTA AGG TCG TTG GAT-3′ |
| Apt-M12 | 5′-CGA GGG ACG TTG CCA ACG GTA ACG TGG TTG GAT-3′ |
| Apt-M13 | 5′-CGG GGG ACG TTG CCA ACG GTA ACG TCG TTG GAT-3′ |
| Apt-M15 | 5′-CGA GGG ACG TTG CCA ACG ATA AGG TGG TTG GAT-3′ |
| FAM-Apt1 | 5′6-FAM ACA CGA CGA GGG ACG AGG AGT ACT TGC CAA CGA TAA CGT CGT TGG ATC TGT CTG TGC CC-3′ |
| FAM-Apt-M13 | 5′6-FAM CGG GGG ACG TTG CCA ACG GTA ACG TCG TTG GAT-3′ |

## Engineering constructed high-affinity aptamer for ultrasensitive colorimetric detection of dexamethasone in raw milk

**Yadi Qin^1^, Yanan Qin^2^, Hayilati Bubiajiaer ^1^, Jun Yao^1^**^[[1]](#footnote-1)^***, Minwei Zhang^2 *^**

*^[1]^School of Pharmacy, Xinjiang Medical University,830054 Xinyi Road Urumqi, China.*

*^[2]^College life science & technology, Xinjiang University, 830046 Shengli Road Urumqi, China.*

Table S1 Nucleotide sequences of truncated and mutated aptamers

Fig.S1 Secondary structures of the original aptamer and truncated aptamers, and flow charts of the truncations. The binding site in the original aptamer sequence is highlighted.

| Table S2 The list of aptamer sequences, the mutated base is represented by X | |
| --- | --- |
| **Name** | **Sequence** |
| Apt1 | 5′-ACA CGA CGA GGG ACG AGG AGT ACT TGC CAA CGA TAA CGT CGT TGG ATC TGT CTG TGC CC-3′ |
| Apt-T1 | 5′-CGA GGG ACG AGG AGT ACT TGC CAA CGA TAA CGT CGT TGG AT-3′ |
| Apt-T2 | 5′-CGA GGG ACG TTG CCA ACG ATA ACG TCG TTG GAT-3′ |
| Apt-T3 | 5′-TTG CCA ACG ATAACGTCGTTGGAT-3′ |
| Apt-S1 | 5′-GGA GGG ACG TTG CCA ACG ATA ACG TCG TTG GAT-3′ |
| Apt-S2 | 5′-CGG GGG ACG TTG CCA ACG ATA ACG TCG TTG GAT-3′ |
| Apt-S3 | 5′-CGA GGG AGG TTG CCA ACG ATA ACG TCG TTG GAT-3′ |
| Apt-S4 | 5′-CGA GGG ACG GTG CCA ACG ATA ACG TCG TTG GAT-3′ |
| Apt-S5 | 5′-CGA GGG ACG TGG CCA ACG ATA ACG TCG TTG GAT-3′ |
| Apt-S6 | 5′-CGA GGG ACG TTG GCA ACG ATA ACG TCG TTG GAT-3′ |
| Apt-S7 | 5′-CGA GGG ACG TTG CCA AGG ATA ACG TCG TTG GAT-3′ |
| Apt-S8 | 5′-CGA GGG ACG TTG CCA ACG GTA ACG TCG TTG GAT-3′ |
| Apt-S9 | 5′-CGA GGG ACG TTG CCA ACG ATA AGG TCG TTG GAT-3′ |
| Apt-S10 | 5′-CGA GGG ACG TTG CCA ACG ATA ACG GCG TTG GAT-3′ |
| Apt-S11 | 5′-CGA GGG ACG TTG CCA ACG ATA ACG TGG TTG GAT-3′ |
| Apt-S12 | 5′-CGA GGG ACG TTG CCA ACG ATA ACG TCG GTG GAT-3′ |
| Apt-S13 | 5′-CGA GGG ACG TTG CCA ACG ATA ACG TCG TGG GAT-3′ |
| Apt-S14 | 5′-CGA GGG ACG TTG CCA ACG ATA ACG TCG TTG GGT-3′ |
| Apt-M1 | 5′-GGA GGG AGG TTG CCA ACG ATA ACG TCG TTG GAT-3′ |
| Apt-M2 | 5′-GGA GGG ACG TTG CCA ACG GTA ACG GCG TTG GAT-3′ |
| Apt-M3 | 5′-GGA GGG ACG TTG CCA ACG GTA ACG TCG TTG GAT-3′ |
| Apt-M4 | 5′-GGA GGG ACG TTG CCA ACG ATA ACG GCG TTG GAT-3′ |
| Apt-M5 | 5′-GGA GGG ACG TTG CCA ACG ATA ACG TCG GTG GAT-3′ |
| Apt-M6 | 5′-CGA GGG AGG TTG CCA ACG GTA ACG TCG TTG GAT-3′ |
| Apt-M7 | 5′-CGA GGG AGG TTG CCA ACG ATA AGG TCG TTG GAT-3′ |
| Apt-M8 | 5′-CGA GGG AGG TTG CCA ACG ATA ACG GCG TTG GAT-3′ |
| Apt-M9 | 5′-CGA GGG ACG TTG GCA ACG GTA ACG TCG TTG GAT-3′ |
| Apt-M10 | 5′-CGA GGG ACG TTG GCA ACG ATA ACG TGG TTG GAT-3′ |
| Apt-M11 | 5′-CGA GGG ACG TTG CCA ACG GTA AGG TCG TTG GAT-3′ |
| Apt-M12 | 5′-CGA GGG ACG TTG CCA ACG GTA ACG TGG TTG GAT-3′ |
| Apt-M13 | 5′-CGG GGG ACG TTG CCA ACG GTA ACG TCG TTG GAT-3′ |
| Apt-M14 | 5′-CGA GGG ACG TTG CCA ACG GTA ACG TCG GTG GAT-3′ |
| Apt-M15 | 5′-CGA GGG ACG TTG CCA ACG ATA AGG TGG TTG GAT-3′ |
| Apt-M16 | 5′-CGA GGG ACG TTG CCA ACG ATA ACG TCG GGG GAT-3′ |
| Apt-M17 | 5′-CGA GGG ACG TTG CCA ACG GTA ACG TCG GGG GAT-3′ |
| Apt-M18 | 5′-CGA GGG AGG TTG CCA ACG GTA ACG GCG TTG GAT-3′ |
| Apt-M19 | 5′-GGA GGG ACG TTG CCA ACG GTA ACG GCG TTG GAT-3′ |

Fig. S2 UV absorption spectra of a sensing system based on the truncated aptamer Apt-T1 at DEX concentrations of 2-350 nmol/mL and linear responses at different DEX concentrations(A). UV absorption spectra of a sensing system based on the truncated aptamer Apt-T3 at DEX concentrations of 2-350 nmol/mL and linear responses at different DEX concentrations(B).

Fig. S3 Secondary structure prediction of the mutant aptamers.

Fig. S4. UV absorption spectra of different mutant aptamer-based sensing systems at DEX concentrations of 2-350 nmol/mL and linear responses at different DEX concentrations.

Fig. S5 The fluorescence spectrum of different aptamers mixed with SGI (100 μL 50 nM aptamer + 15 μL 1x SGI + 385 μL ddH_2_O)

1. * * Corresponding authors. Tel :18999250641 (J, Yao); 13999258239（M, Zhang）

   E-mail addresses: [xydyaojun@163.com](mailto:xydyaojun@163.com) (J, Yao); [zhangmw@xju.edu.cn](mailto:zhangmw@xju.edu.cn) (M, Zhang) [↑](#footnote-ref-1)
